# Blood Clot Simulation Model by Using the Bond-Graph Technique

**DOI:** 10.1155/2013/519047

**Published:** 2013-12-22

**Authors:** Gregorio Romero, M. Luisa Martinez, Joaquin Maroto, Jesus Felez

**Affiliations:** CITEF, Universidad Politecnica de Madrid, C. Jose Gutierrez Abascal 2, 28006 Madrid, Spain

## Abstract

The World Health Organization estimates that 17 million people die of cardiovascular disease, particularly heart attacks and strokes, every year. Most strokes are caused by a blood clot that occludes an artery in the cerebral circulation and the process concerning the removal of this obstruction involves catheterisation. The fundamental object of the presented study consists in determining and optimizing the necessary simulation model corresponding with the blood clot zone to be implemented jointly with other Mechanical Thrombectomy Device simulation models, which have become more widely used during the last decade. To do so, a multidomain technique is used to better explain the different aspects of the attachment to the artery wall and between the existing platelets, it being possible to obtain the mathematical equations that define the full model. For a better understanding, a consecutive approximation to the definitive model will be presented, analyzing the different problems found during the study. The final presented model considers an elastic characterization of the blood clot composition and the possibility of obtaining a consecutive detachment process from the artery wall. In conclusion, the presented model contains the necessary behaviour laws to be implemented in future blood clot simulation models.

## 1. Introduction

The World Health Organization reports that 15 million people worldwide suffer strokes [1] and of these, 5 million die and further 5 million are left permanently disabled, many severely impaired. Cardiovascular diseases (CVDs) are the primary cause of death globally. It is estimated that more than 15 million people died from this cause in 2004, which represents a very important percentage of all global deaths; this amounts to an estimated 5.5 million deaths due to stroke. In the United Kingdom alone there are 130,000 strokes each year and of the patients that survive many are left with severe disabilities. Strokes are devastating events, 85% of which are caused by the blockage of an artery that prevents blood from flowing to the brain.

Atheromatous plaques are a common cause associated with the narrowing or blockage of an artery. These plaques arise from a build-up of fatty deposits on the inner walls of the blood vessels. This results in the formation of an atheromatous plaque that narrows the artery that it is located in. Parts of the plaque may break away and travel to the brain blocking a vessel or blood clots that arise from the alteration of blood flow dynamics associated with the narrowing of the vessel can cause a stroke. Generally speaking, changes in blood flow may arise due to a change in the composition of the blood or a change in the blood flow itself (e.g., due to an uneven surface within the vessel wall) such as would be created by plaque formation. Such a change in blood flow predisposes to clot formation. Blood clots arise when platelets adhere to each other and to the artery wall. A clot may therefore readily form at a site where a vessel is narrowed.

Although thrombolytic agents such as alteplase are used to dissolve blood clots that arise in the cerebral arteries of the brain, there are limitations to the use of such thrombolytic agents. During the last decade Mechanical Thrombectomy Devices (MTDs) have become more widely used as an alternative means for clot removal. A number of devices using a variety of methods to remove the clot are now available. These include the MERCI clot retriever, and, more recently, the penumbral device; other types of devices include angiography catheters, rheolytic catheters (Angiojet), Basket style devices, and microsnaring devices. Thrombectomy may be associated with risks, such as breakage of moving parts, penetration of the arterial wall, and downstream embolisation caused by clot dislodgment [2]. Studies suggest that mechanical embolectomy is most effective in large volume proximal occlusions [3]. Other interventional surgical treatments include endarterectomy, which involves surgically removing clots in the carotid arteries. This treatment has proved successful [4] but carries a risk of the clot becoming dislodged during the procedure.

New designs of Thrombectomy Devices to remove blood clots without the need to make contact with the clot itself, thereby potentially reducing the risk of problems such as downstream embolisation, for example, by using aspiration techniques, need to be studied and analyzed in order to obtain better results. The analysis, design, and optimization of one experimental device recently developed in the UK, called the “GP” Thrombus Aspiration Device (GPTAD) [5–10], has been recently done by using different simulation techniques [11, 12].

During the development of potential new medical devices computer premodelling may be required to help in the optimization and fine-tuning of the devices. The main objective of this simulation model is to obtain the characterization of a blood clot model considering the interaction between the blood clot and artery wall and the behaviour laws.

Next, the model used for the simulation is consecutively described as well as the phenomena considered, and, in addition, the values of the parameters used are defined.

## 2. Initial Blood Clot Approximation

Accurately defining the clot model in order to correctly simulate a full aspiration thrombectomy device interaction is the most complex part of the model. A clot is a cylindrically shaped element of 1.0–5.0 cm long, and a mass that falls between 0.5 and 2.0 gram. This element has usually formed in another location, usually in a vascular artery, and has become dislodged remaining trapped in smaller diameter arteries, such as the cerebral artery considered in this work. Therefore, due to this difference in diameter, the clot becomes attached to the wall by a force that needs to be overcome in order to begin its movement for removal. In addition, the relative movement between the clot and the artery presents static and dynamic friction, which needs to be taken into account. If a correct approximation to reality is to be achieved all these phenomena, as well as the circumstances restricting clot movement, need to be considered.

As already stated, the phenomenon preventing clot movement is the difference in diameter between the clot and the artery where it is located. Experimental data [5] and numerical studies [13] indicate that the clot begins its movement when this force is equal to 0.01 N. In order to decide when to begin the movement of the obstructive element, firstly we need to insert a spring into the model to measure the force supported by the beginning of the clot and its deformation. To obtain the value of this spring the phenomenon of surface tension must be taken into account, since it is this that joins the clot to the artery. This surface tension “*γ*” comes about from the attraction forces between molecules and is defined as force by unit length:

(1) γ=Fl.

**Figure 1:**
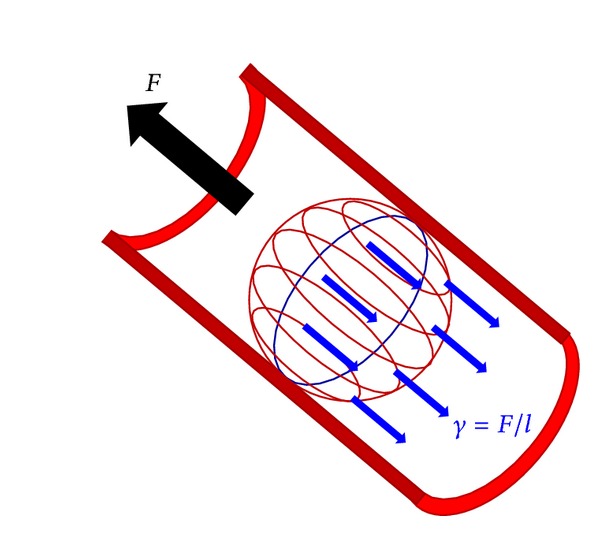
Surface tension (blue circle).

If we take the sphere shown in Figure 1, the surface tension would act on the circumference of the contact between the clot and the artery (marked with a blue ellipse).

Bearing in mind the above, if we take into account the value of the length of contact “*l*” from the radius “*r*” of the artery, the value for the surface tension “*γ*” can be obtained and, in turn, the value for the spring coefficient “*K*”, as

(2) K=γ=Fl.

**Figure 2:**
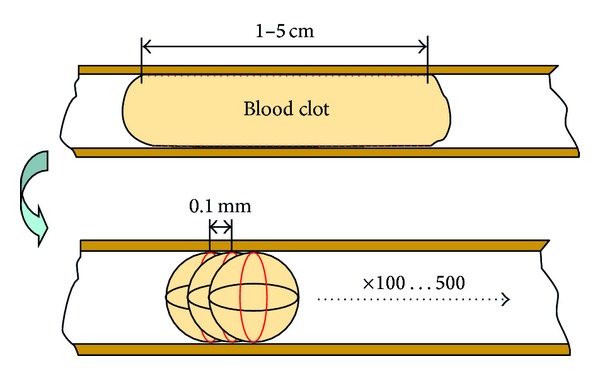
Surface tension from some spheres.

As stated above, the clot is 1.0–5.0 cm long, which means it can be broken down into the union of several spheres (Figure 2), all with the same constant. Since over the whole surface of the clot there are adhesion forces, to obtain a correct approximation it is necessary to consider the existence of a sphere for every 0.1 mm. This means that between 100 and 500 spheres would need to be included in the model. On the other hand, since the clot behaves like a rigid body, the fact that all the spheres are located in parallel must be taken into account, so that until the resultant adhesion force is overcome in all of the spheres, the clot will not begin to move.

Therefore, since all the individual springs are equal, the equivalent spring value “*K*_eq_” for a determined clot length can be obtained considering the following expression

(3) Keq=Kn,

where “*K*” is the spring coefficient determined in (2) as the surface tension and “*n*” is the number of elements considered taking into account the existence of one sphere for every 0.1 mm length.

To know when the force will be reached in this equivalent spring and, therefore, when the clot movement will begin, it is essential to calculate the displacement of the spring when it is subjected to 0.01 N through a typical spring equation. Therefore, only when the spring undergoes this displacement should the clot be allowed to move; to the contrary it would be prevented:

(4) x=FKeq.

**Figure 3:**
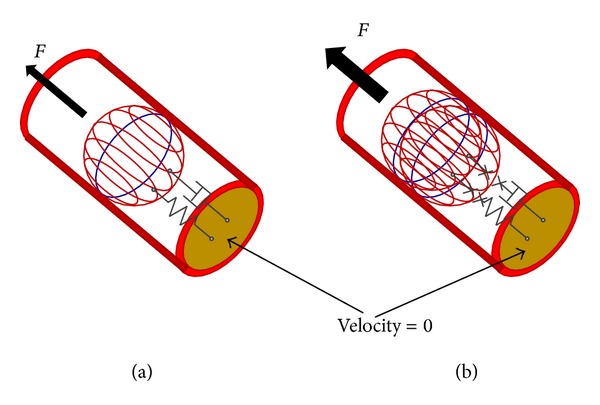
Spring-damper system. (a) *F* < 0.01 N. (b) *F* ≥ 0.01 N.

Secondly, it is necessary to insert the resistance that represents the friction between the clot and the arterial wall. The value of this parameter must be variable depending on whether the clot has not begun its movement (static friction) or if it is already in movement (dynamic friction), so that when the clot begins to move the friction value will drop considerably (Figure 3). This value is obtained starting from the Stokes equation and can be given a value of 2.5 · 10^−6^ N·s/m for the static friction and an order of magnitude lower than for the dynamic friction.

The transition between both values marks the beginning or end of the clot movement by means of the displacement of the spring representing the deformation described in expression (4). So, when the displacement undergone by this spring is less than that calculated, static friction will rule; to the contrary, if it is greater, the friction will be dynamic.

In addition to the spring and the resistance inserted, the model must have an inertance that represents the mass of the blood clot (0.5–2.0 gram):

(5) I=m.

Finally, to ensure that the clot remains at rest while the force existing at its beginning is less than 0.01 N, a spring-damper system joined to a wall (zero flow source) must be used.

In this system, while the clot does not receive the force of minimum suction, it has a zero speed. However, when it begins its movement, the spring-damper system must be cancelled allowing its extraction. While the force representing the deformation of the clot is lower than 0.01 N, it will remain attached to the wall, thereby preventing any movement. To the contrary, if the force exceeds this figure the model will cause the bond imposed by the spring-damper system to be eliminated with the clot becoming free and moving in accordance with the suction pressure acting on it from the system, letting it be removed.

Therefore, the elimination of the spring-damper system will be performed from the displacement of the spring representing the clot deformation as was seen in the condition imposed on the static/dynamic friction. The initial values for the spring-damper system have been considered, 1 · 10^10^ N/m and 1 · 10^9^ N·s/m, respectively; these values have been taken to consider a very rigid union and the correct convergence of the system.

**Figure 4:**
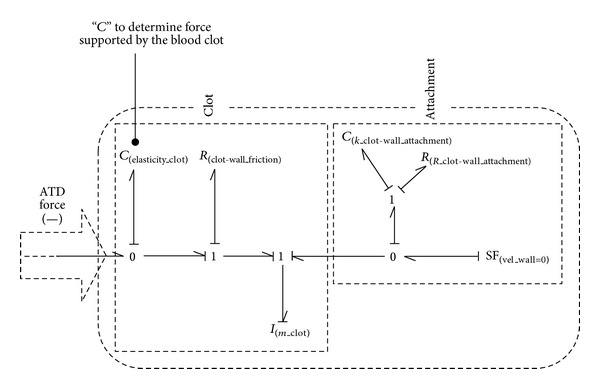
The simplest blood clot model by Bond-Graph technique.

Figure 4 shows the Bond-Graph scheme of the different elements commented below, having indicated where the aspiration force created by the Aspiration Thrombectomy Device (ATD) would be added.

The main problem of this initial model is that the blood clot is fully considered as a simple element, while the possibility of partial disgregation does not exist. In addition, this model is able to simulate the behaviour of the blood clot attachment but the values of the necessary compliances and resistances introduced can only be estimated from different experiments. The value of the constants in both the spring and the damper must be extremely high to simulate a firm anchor to the point of release. Simulating the moment when the clot breaks loose from the wall in the previous model method was very difficult, because the junction to the wall had to allow clot movement according to pressure, even if such pressure was not enough to move it in totality. In addition, low value changes introduced in these elements can modify substantially the pressure and time results.

The previous comments are the main reason why a better approximation model concerning the blood clot and artery wall attachment needs to be studied.

## 3. Extended Blood Clot Approximation

**Figure 5:**
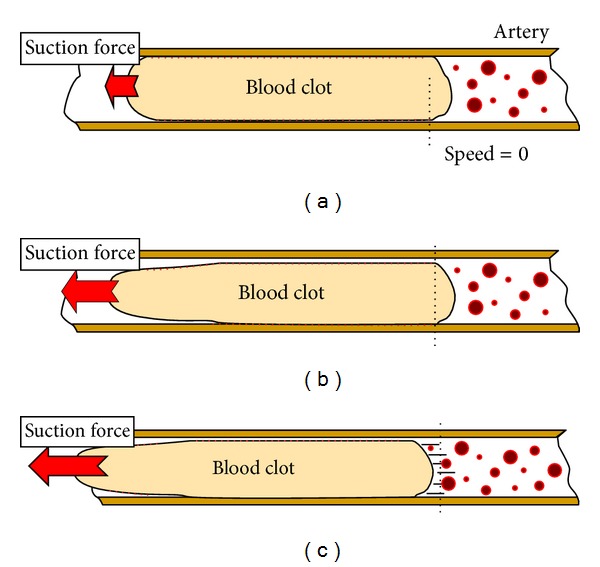
Influence of the suction force over blood clot. (a) The lowest value; no movement. (b) Medium value; beginning of the front part movement. (c) Higher value; blood clot movement.

For a correct understanding of the blood clot detachment process (see Figure 5), it is necessary to take into account that the friction between the clot and the arterial wall creates a resistance factor, not over all the surface, but incrementally from the beginning to the end.

By considering this, it is possible to understand that the value of this parameter must be variable across the length depending on whether the clot has begun its movement (Figure 5(a)) or before it has begun to move (Figures 5(a) and 5(b)) during the clot extraction procedure. To do this, an extended blood clot will incorporate different inertia elements representing different partitions of the blood clot, joined by different spring-damper systems (designed by *K*_union_ and *R*_union_, resp.) between them to simulate the elastic and plastic behaviour of the clot, and the consequent spring-damper systems joined to the wall, which could be defined by a zero flow source.

When the clot begins to move the friction decreases considerably. In the same way as previously, this value is obtained from the Stokes equation and can be given a value of 2.5 · 10^−6^ N·s/m for the static friction and an order of magnitude lower than for the dynamic friction; the transition between both values marks the beginning or end of the clot movement.

**Figure 6:**
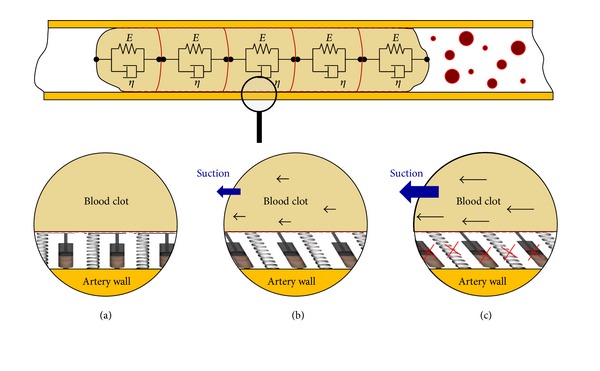
Artery wall adherence based on individual spring-damper systems. (a) No force. (b) Suction force lower than adherence force. (c) Suction force higher than adherence force.

The existence of the different partitions (Figures 5 and 6), and the consequent spring-damper systems joined to the wall, let the model acquire a more realistic behaviour due to the fact that the different inertias detachment can be treated individually.

### 3.1. Platelet Composition Model

**Figure 7:**
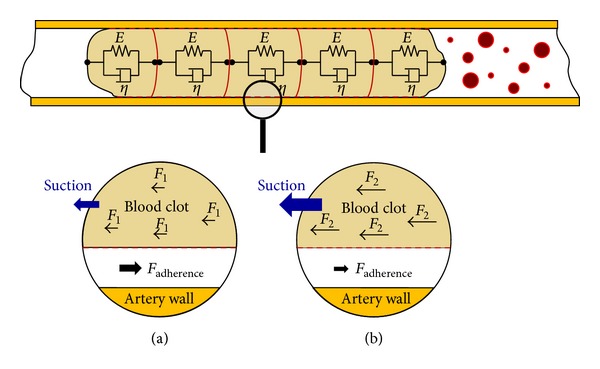
Adherence and friction based on effort source. (a) Lower suction force; no movement. (b) Suction force higher than adherence force.

Although the extended clot approximation is necessary, due to the problems presented in the previous model, it is necessary to look for another solution to simulate the clot and its behaviour under a negative force. As we have seen before, we kept the partitions represented by inertia and joined by the spring-damper system (*R*_union_, *K*_union_) to simulate the elastic and plastic behaviour of the clot (Figure 7).

**Figure 8:**
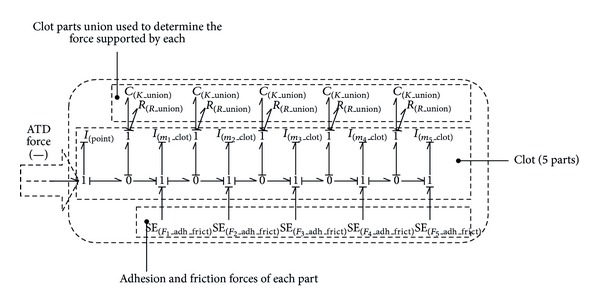
The simplest blood clot model by Bond-Graph technique.

Nevertheless, to model the junction with the artery, the point of release, and the static and dynamic friction, we decided to add to each inertia an effort source that varies depending on the moment of the simulation. We can observe the new configuration in Figure 8. In this figure, it is possible to observe that each inertia will suffer a force due to suction, which should be compensated in the model with a force of friction to annul it while the clot is in the position of static friction. Once we have calculated the flow-effort table of the system, we apply the condition that the stress on the inertia must be zero.

The effort source, as we have stated before, varies; that is, when the clot begins its movement, the static friction disappears and the dynamic friction acts on the system, which is lower than the previous one. We have calculated it by means of the Stokes law for a cylindrical solid (see (7)):

(6) Fdynamic_friction=C8·ρ·π·D2·V2,

where “*C*” is the form coefficient for a cylinder, “*ρ*” is the blood density, “*D*” the clot diameter, and “*V*” the velocity of the first partition.

The condition to determine if the clot is attached to the surface is based on the force that the spring suffers between partitions (*K*_union_). Hence when *K*_union_ *·*Displacement(*K*_union_) is higher than the adherence force, the clot releases from the surface.

**Figure 9:**
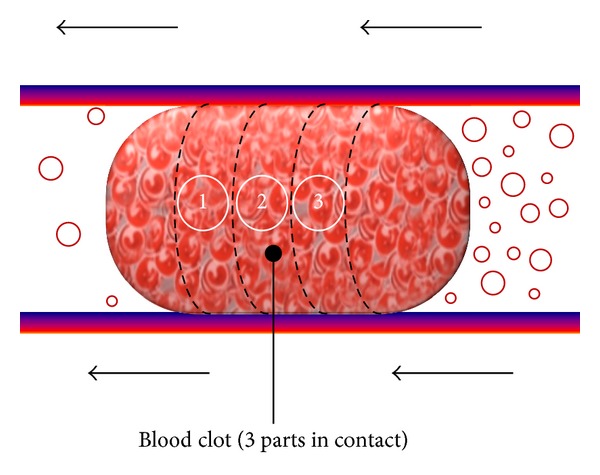
Blood clot 3 parts division.

The value of the adherence force has been analyzed by Flannery [14] and we made use of all the necessary data from that. To calculate the adhesion strength Flannery divided the blood clot into some parts; this geometry is only valid for the condition of static friction, because, once the clot releases, it recovers a part of its form and again becomes like a cylinder. So, we assumed this model and we divided this clot into 3 parts (Figure 9).

As we have defined our clot, we then calculate the adhesion force by means of the platelet adhesion force; we have obtained from Flannery the equivalent number of platelets per area equation:

(7) $$\begin{array}{c} \text{No.}\;\text{platelets}\;\text{per}\;\text{area}=fp\cdot \frac{\text{SA}}{\text{MPA}}, \end{array}$$

where “*fp*” is the % of platelets in the clot, “MPA” the mean platelet area, and “SA” is the area of the surface in contact with the artery, which can be obtained from the clot length, the number of partitions (3 in the case shown), and the artery diameter.

Once we have obtained the number of platelets in contact with the artery and the force platelet-artery wall, we can calculate the adhesion force of each partition and obviously of the entire clot:

(8) $$\begin{array}{c} F_{\text{adh}}=\text{No.}\;{\text{platelets}}_{\text{TOTAL}}\cdot F_{\text{platelet-}{\text{artery}}_{\text{wall}}}. \end{array}$$

In this way, we would have different adhesion forces depending on the form and size of the clot, and we can reference those to the artery diameter and the percentage of occlusion.

### 3.2. Atheroma Plaque Implementation

**Figure 10:**
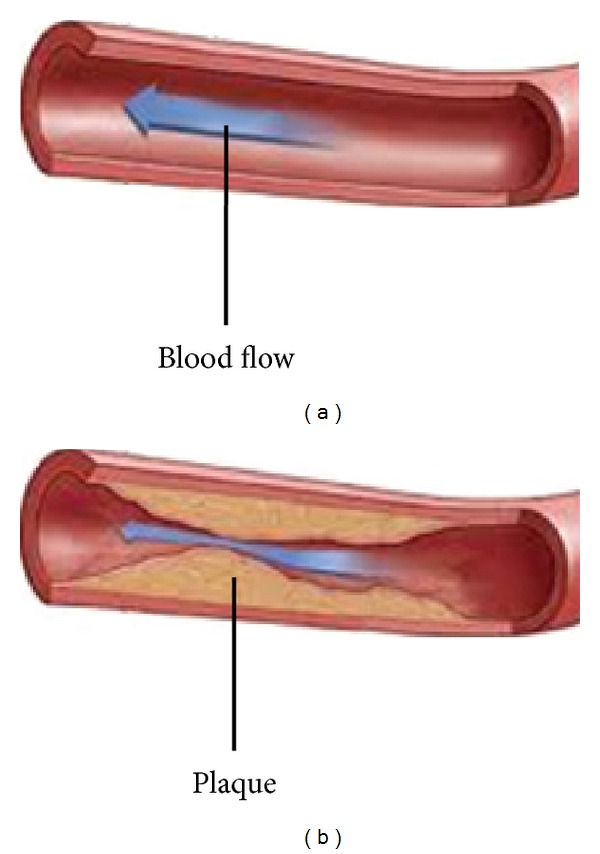
Artery blood flow. (a) Normal artery; (b) Artery with atherosclerosis.

In pathology, an atheroma is an accumulation and swelling in artery walls that is made up of (mostly) macrophage cells, or debris that contain lipids (cholesterol and fatty acids), calcium, and a variable amount of fibrous connective tissue (Figure 10).

**Figure 11:**
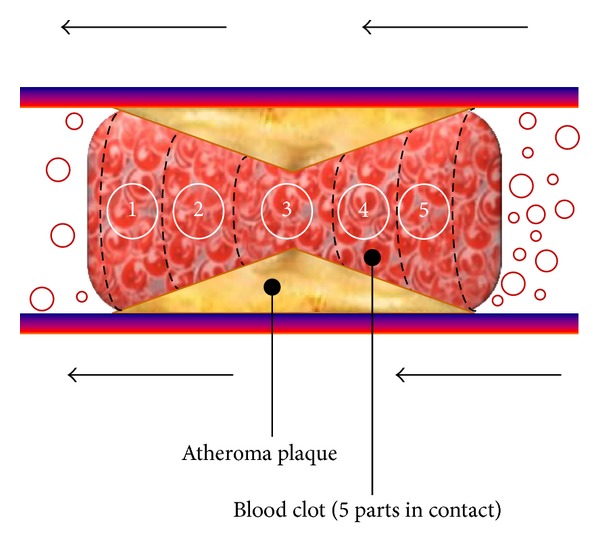
Blood clot 5 parts division.

This accumulation modifies the geometry of the blood as Figure 11 shows and the clot can be considered as a cylindrical clot that narrows down the middle due to stenosis or an atherosclerotic plaque. We decided to make the partitions shown because each one has the same contact area with the artery and the same volume, in order to simplify the calculation of the adhesion force and the mass of each partition. To be more coherent, when the blood clot begins movement each section should be readapted and the mass and contact area recalculated according to Figure 11.

The implementation of the final blood clot model required for the simulation has been made by connecting the five partitions shown in this section and considering the different adhesion and friction forces, jointly with the platelet-platelet forces, which are represented by the *K*_union_ and *R*_union_ elements; this is shown in Figure 9.

### 3.3. Systolic and Diastolic Blood Pressure

For a correct implementation of the blood clot model, it is necessary to implement the systolic and diastolic blood pressure as results of the cardiovascular system.

**Figure 12:**
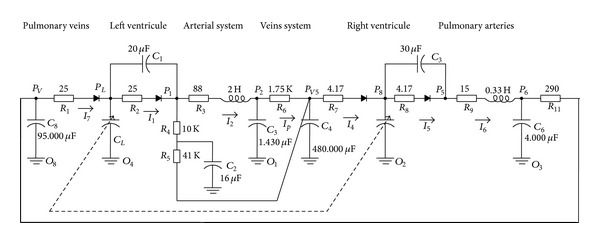
Analog circuit for human systemic circulatory system.

One of the options that has been simulated jointly with the presented model has been that corresponding to the existing analog circuit developed by Noordergraaf at 1978 [15] for the human systemic circulatory system (Figure 12).

Although the simulation of the previous circuit by using Bond-Graph technique is extremely easy, the drawback found was that associated with the diode simulation, since it was observed that, depending on the algorithm used to solve it and the step values, sometimes it did not work properly.

**Figure 13:**
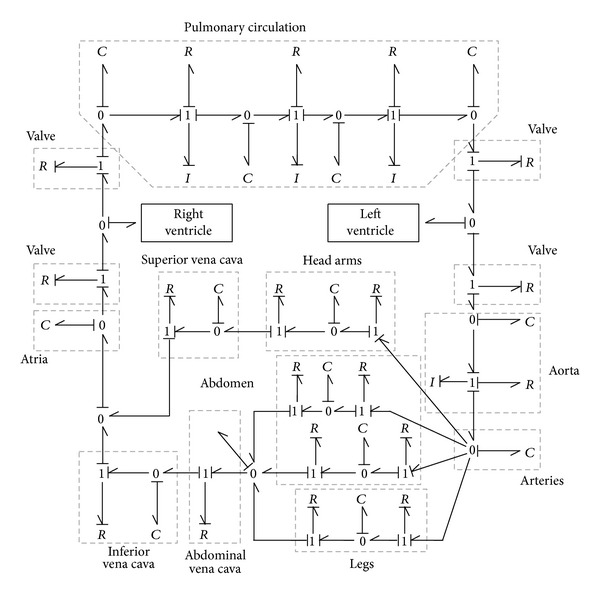
Bond Graph model of the circulation.

Another existing model studied previously by using Bond-Graph technique and valid to be implemented jointly with the presented model in this paper is the one developed by Le Rolle et al. in 2005 [16], which enables the pulmonary and systemic circulation on the head, abdomen, and legs to be analyzed (Figure 13).

Nevertheless, for the analysis of the good performance associated with the presented blood clot model, the systolic and diastolic blood pressure has been introduced only by using a variable pressure.

If we consider that the blood pressure varies from about 120 mmHg to 80 mmHg (16 kPa to 11 kPa) in systolic to diastolic pressure variation in the normal cardiac cycle and we impose a rate of 1 cycle per second, we can approximate mathematically the pressure (kPa) in two parts (0.00–0.32 sec. and 0.32–1.00 sec.) by using the following polynomial expressions:

(9) Pa=13415·t5−8508.6·t4+986.58·t3+177.3·t2−4.99·t+11.01,

(10) Pb=−1488·t6+6237.6·t5−10700·t4+9595.3·t3−4719.5·t2+1188.3·t−102.8.

These equations have been obtained by taking different points from the typical pressure waveform associated.

**Figure 14:**
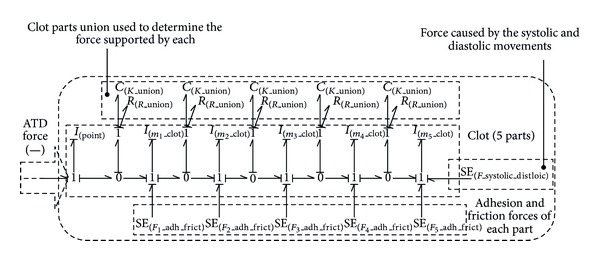
The simplest blood clot model by Bond-Graph technique.

Figure 14 shows the result of implementing the force associated with the previous systolic and diastolic pressure over the 5 partitions model.

## 4. Bi and Tri-Dimensional Blood Clot Model

The different modifications presented from the initial model obtain a good performance of the blood clot over determined situations, as the results will show. Nevertheless, when the intention of the model is to determine, not when the blood clot begins to move or break, but the detaching process platelet-by-platelet, the presented model is not enough.

To do this, the only option is to amplify the partitions, at least in a second dimension. By doing so, the first line of partitions (as shown in the presented model) would be related to each other (platelet-platelet), with the artery wall (platelet-atheroma) and with the second line of partitions (platelet-platelet); in a similar way, each platelet of the second line would be related to each other and with the third line of partitions, obtaining finally a large amount of related partitions (or platelets).

Nevertheless, the main problem associated with the Bond-Graph technique is concerned with each partition (or platelet), which means one differential equation for each partition or each compliance, it not being possible to approach the problem in the event of excessive increase.

To do so, the best way would be to use Agent Based Systems, (ABS) [17], which have emerged as one of the most important areas of research and development. An ABS system is one composed of multiple interacting components known as agents that reason logically in a similar way to presented here.

**Figure 15:**
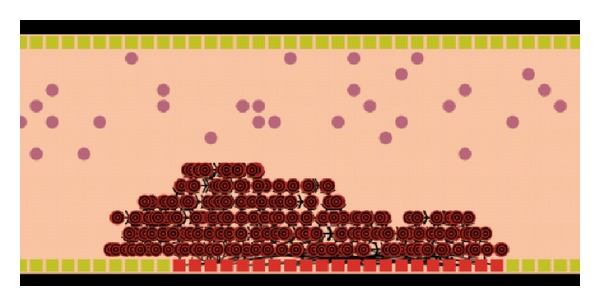
ABS blood clot model (200 agents).

Figure 15 shows a sample made by the authors by using NetLogo software [18]; in this case, it corresponds to 200 partitions, divided into 6 lines. As can be observed, each partition is joined to the closest partitions in a similar way to that presented in this paper.

## 5. Results

As a final result of the presented Bond-Graph model, the aim of this simulation is to determine the time and pressure required for the extraction of a blood clot. To do this, by varying the values of the pressure source, the movement of the clot and the time required for its extraction are measured, thereby obtaining the optimum minimum pressure. To carry out the model validation, the values of the parameters used in the simulation are listed in Table 1.

The parameters that define almost completely the elastic-plastic behaviour of the clot and its resistance to breaking are the constants “*K*_union_” and “*R*_union_” of the spring-damper systems in parallel that are among the partitions of the clot that characterize the clot in the stretch mode, when it undergoes suction but is not yet detached from the wall. To find the value of the “*K*_union_” parameter, Savushkin [19] analyzes the stiffness of the clot and the breaking strength. We considered that the values are valid, due to the fact that the parameters of the experiments described fall within our range, and therefore we can assume that *K*_union_ = 3.41 ± 1.5 N/m.

Concerning the “*R*_union_” value, Pennati et al. [20] considers some useful parameters. In that work, values of the viscosity of the blood appear for the clot that they use in their model; taking into account the viscosity of the clot, we can assume that *R*_union_ = 0.035 kg/m·s.

In the model simulated in this section, we take a blood clot of 2.5 mm in diameter and 1.0–5.0 cm in length. The existence of different partitions in the clot makes the extraction progressive with increasing time.

The existence of different clot lengths will affect the mass of the clot being removed (up to 2 gr.) and increase the time taken for clot removal as the value of the clot length increases. It has been found, that the greater the rigidity of the clot, the shorter the extraction time. This factor is also related to the viscosity and composition of the clot that will vary in each case. Therefore, for this study we have determined the critical values possible, making the assumption that there are 96% platelets in the clot, which yields a fairly high bond strength, which gives us an idea of the maximum pressure needed.

**Figure 16:**
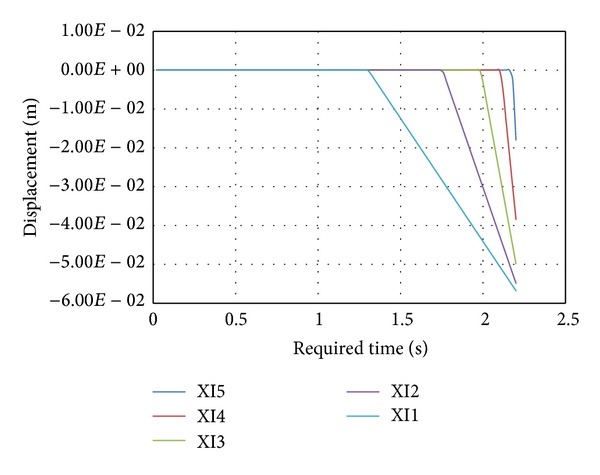
Movement of each blood clot part.

Figure 16 shows the movement of each blood clot taking into account the increasing value of the suction pressure in the first second. Once the force over one partition is greater than the adhesion force in it between blood clot and artery, the adhesion is broken and clot movement begins. In addition, it is possible to look at the velocity when one section is detached and this is higher since all the force is applied over lesser partitions.

**Figure 17:**
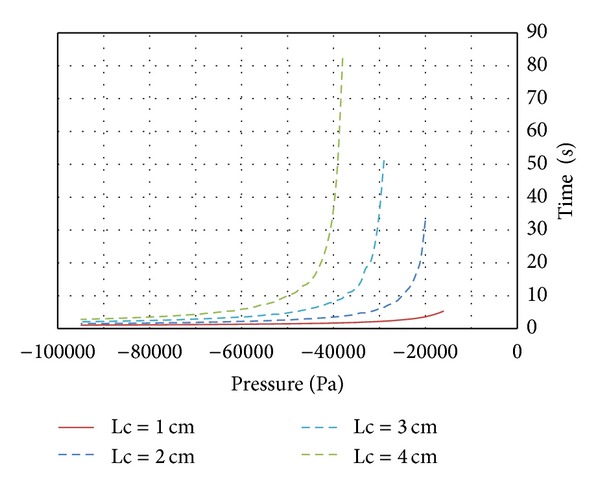
Required depressure versus time.

The usefulness of the presented model is concerned with the time the blood clot takes to move a concrete distance (e.g., 2-3 mm), which is the distance that is maintained during the extraction to the TAD device. In Figure 17, we can observe the different times to extract a blood clot in a 100% occlusion case, with a diameter of *D* = 2.5 mm and for different lengths such as 1.0–5.0 cm, respectively; these results have been compared with those obtained by using the GP at the laboratory [5] and it contains very similar results.

As we can see, the lower the length the lower the time needed to extract the clot, because we have less adherence force and less inertia due to the mass. We have applied it to the extraction of a 5.0 cm length but there is a danger of rupture prior to complete clot removal, which would mean the failure of the process, due to the fact that the force supported by the blood clot is higher than the rupture force (more length means more adherence).

## 6. Conclusions

We have studied the formation, composition, and shape of different blood clots in different cases, trying to find some general parameters that could define their behaviour reliably. In particular, we have studied the influence of composition, form, and some parameters that directly affect the adhesive force that holds the clot against the arterial wall. The most significant inclusion in the model, and therefore the one that provides greater reliability to the model, has been the change in the approach regarding the internal structure of the clot and its adherence to the wall.

It is necessary to say that, for the exact modelling of the laboratory experiment, the different parameters should have been obtained over the existing blood clot, not from the literature.

Many previous studies analyze similar models by using Finite Element Analysis, where it is often necessary to reproduce the full situation which usually requires a lot of computer simulation time. Nevertheless, there is not any model developed by using the Bond Graph technique.

Apart from demonstrating that the technique is very useful to represent the different simulation conditions, making it possible to incorporate different parameters in a very effective straightforward manner, the very simple model obtained in the presented work will let future researchers obtain the differential equation system, without the need of complex models by using Finite Element Analysis or Agent-Based Systems techniques.

## Figures and Tables

**Table 1:** Parameter values.

Blood density (*ρ*)	1060 Kg/m^3^
Artery diameter	2.5 mm
Blood viscosity (*η*)	0.0035 Pa·s
*K*_union_	1.91 N/m
*R*_union_	0.035 N·s/m
Clot length	(1.0–5.0) cm
fp	0.96
MPA	5.31 · 10^−6^ mm^2^
*F*_adhesion_platelet_	32 · 10^−9^ N
Occlusion	100%
